# Complete Genome Sequences of Rhizobium gallicum M101 and Two Potential New *Rhizobium* Species Isolated from Soils in Central Canada

**DOI:** 10.1128/mra.00216-22

**Published:** 2022-06-07

**Authors:** Justin P. Hawkins, Patricia A. Ordonez, Ivan J. Oresnik

**Affiliations:** a Department of Microbiology, University of Manitoba, Winnipeg, Manitoba, Canada; University of Arizona

## Abstract

Here, we report the genome sequences of Rhizobium gallicum M101, *Rhizobium* sp. strain C104, and *Rhizobium* sp. strain K102. These bacteria were isolated from three locations in Manitoba, Canada. The M101 genome meets the criteria for R. gallicum based on average nucleotide identity and DNA-DNA hybridization; the genomes of C104 and K102 are below the thresholds to be matched to known type strains.

## ANNOUNCEMENT

In Canada and the northern United States, inoculants used for cultivars of Phaseolus vulagaris form ineffective nitrogen-fixing relationships. To create an effective inoculant, it was hypothesized that bacteria that form nodules on P. vulgaris and are present in the soil in these areas would be ideal targets for adaptation to become nitrogen-fixing bacteria. Bacteria that fit this criterion were isolated from soil in Melita, Carman, or Kelburn (Manitoba, Canada). Here, we report the isolation of a new strain of Rhizobium gallicum and two potentially new *Rhizobium* species.

Nitrogen-fixing nodules were isolated from plant roots, surface sterilized with 1% bleach, washed with sterile distilled water, and finally crushed in a microcentrifuge tube containing 50 μl of water. Bacteria were plated from this suspension on tryptone-yeast extract (TY) agar (1), and individual colonies were streaked three times to purity. Bacteria were cultured in TY broth for 2 days, and genomic DNA was isolated using the PureLink genomic DNA minikit (Invitrogen). Sequencing was carried out using a Nanopore MinION Mk1B system with SQK-LSK-109 and EXP-NBD-104 kits and R10.3 flow cells, and base calling was handled by Guppy-GPU (2). Default parameters were used for all software in the analysis. Approximately 50,000 reads were trimmed using BBduk (3), and then *de novo* genome assembly was carried out using Flye, followed by three rounds of polishing using minimap2 (4, 5). Genome completeness was estimated using CheckM (6) and was found to be above 99%, with an estimated 1% contamination in all three assemblies. The genome assembly of Rhizobium gallicum M101 resulted in 3 circular contigs with a total size of 7.26 Mbp, a GC content of 59.7%, and coverage of 95×. *Rhizobium* sp. strain C104 showed 8 circular contigs and 1 linear contig with a total size of 7.55 Mbp, a GC content of 61.0%, and coverage of 188×. *Rhizobium* sp. strain K102 was assembled into 7 circular contigs with a size of 6.74 Mbp, a GC content of 61.4%, and coverage of 50×. Genomes were annotated using the Prokaryotic Genome Annotation Pipeline (PGAP) (7).

**FIG 1:**
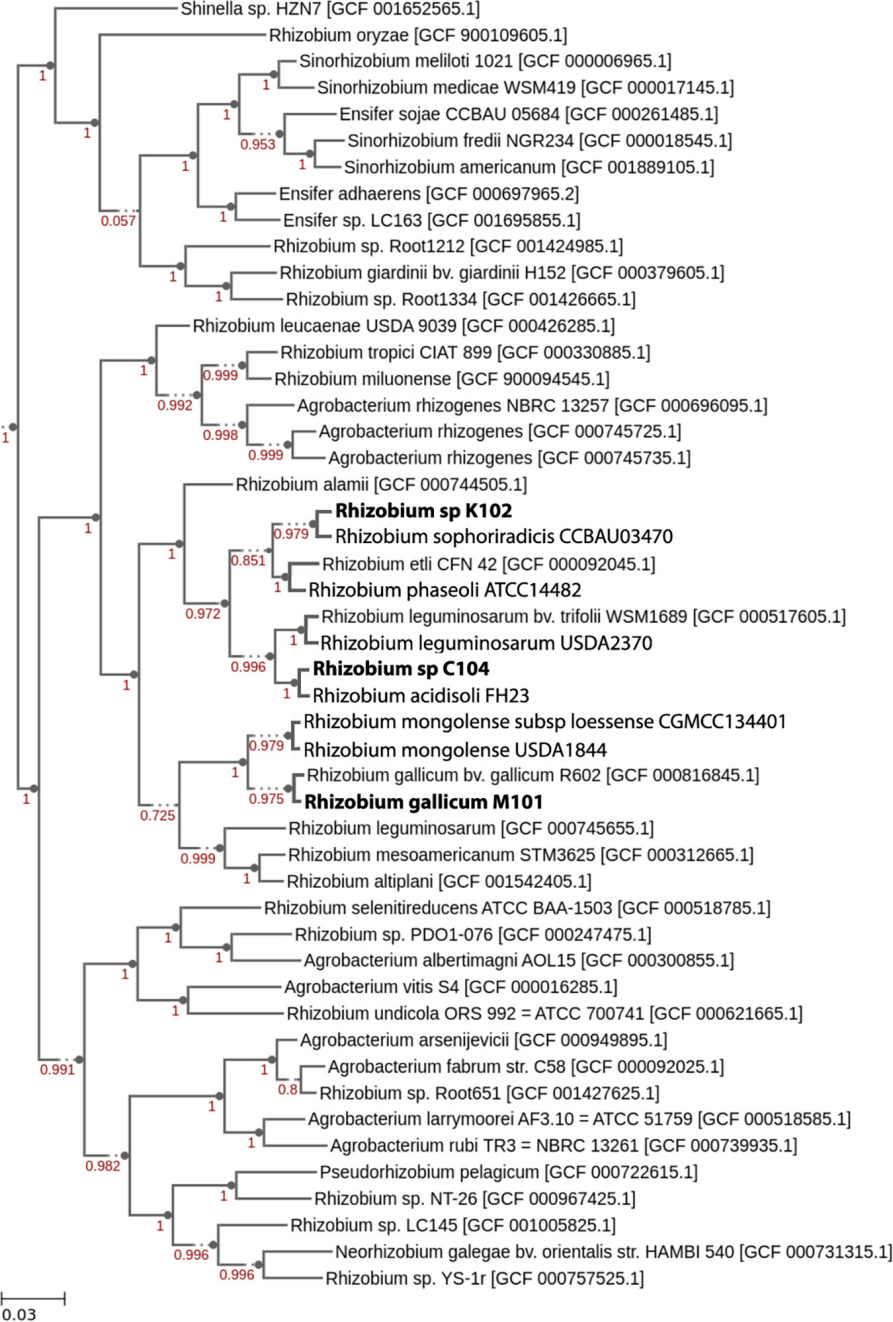
Phylogenetic tree of sequenced rhizobial strains. The phylogeny was created using the SpeciesTreeBuilder tool in the KBase environment running FastTree2. This tree represents a phylogenetic analysis of 49 conserved core genes from the strains most closely related to the query genomes. Branch lengths and node support were calculated by the program, and the scale represents amino acid substitutions per site. The top two hits based on ANI for each query were inserted into the tree when not represented by default by the program. The dotted lines indicate a split with less than 100% bootstrap support. Accession numbers for each genome is indicated in brackets.

The 16S rRNA gene was compared against those of known type strains using BLASTn (8) with the EzBioCloud server (9), followed by examination of all identified type strain genomes using average nucleotide identity (ANI) values determined with fastANI (10) and digital DNA-DNA hybridization (dDDH) through the Type Strain Genome Server (TYGS) (11). The closest related species for each query can be seen in Table 1. To determine the closely related species for R. gallicum M101, FastTree2 (12) was used to construct a phylogeny of closely related species through SpeciesTreeBuilder v2.2.0 in KBase (13) (Fig. 1). M101 was observed to cluster with R. gallicum R602, separate from the other two closely related species. Both *Rhizobium* species strains clustered with the species determined to be the closest related species from fastANI and dDDH.

**TABLE 1:** ANI and DNA-DNA relatedness to closely related strains

Strain	No. of reads (×1,000)	No. of base pairs sequenced	*N*_50_ (kbp)	Comparison strain	ANI (%)	16S rRNA identity (%)	dDDH (%)			GC content difference (%)
							Formula 1	Formula 2	Formula 3	
R. gallicum M101	56.7	0.7 Gbp	23.7	R. gallicum R602^T^	95.60	99.40	58.40	68.00	61.30	0.06
				Rhizobium mongolense USDA1844^T^	95.60	99.60	57.50	68.20	60.50	0.17
				R. mongolense subsp. *loessense* CGMCC1.3401^T^	95.20	99.60	62.60	64.40	64.60	0.04
Rhizobium sp. strain C104	124.5	1.4 Gbp	21.2	Rhizobium acidisoli FH23	94.09	99.93	66.90	55.50	66.30	0.06
Rhizobium sp. strain K102	127.2	1.3 Gbp	20.3	Rhizobium sophoriradicis CCBAU3470^T^	93.63	99.64	77.80	52.10	74.50	0.12

Using Mauve to align genomes (14), it was found that R. gallicum M101 has 84.5% identity in the *nif, nod*, and *fix* regions and harbors the same genes as R. gallicum R602 (Accession number GCA_000816845.1). This region from K102 showed 98% identity with the same region from Rhizobium etli CFN42^T^ (GCF_000092045.1) but contained a potential duplication of the *nolO* and *nifB* genes. In strain C104, this region displayed 85% identity to that of Rhizobium acidisoli FH23 (GCA_002531755.2) and contained the same genes except for missing *nodZ* and an insertion of *nolO*.

### Data availability.

The genomes have been deposited under BioProject accession number PRJNA798730 and BioSample accession numbers SAMN25122528, SAMN25861395, and SAMN25942628. Genome assembly and Sequence Read Archive (SRA) accession numbers are as follows: R. gallicum M101, GCA_022354485.1 and SRR18192451; *Rhizobium* sp. strain C104, (GCA_022354505.1 and SRR18192449; *Rhizobium* sp. strain K102, GCA_022385315.1 and SRR18192450).
